# Case report: Bilateral carotid body tumors with a concomitant skull-base paraganglioma

**DOI:** 10.3389/fonc.2023.1120152

**Published:** 2023-03-21

**Authors:** Zhixuan Liu, Ruimin Yue, Cuiyun Sun, Junping Wang

**Affiliations:** ^1^ Department of Radiology and Tianjin Key Laboratory of Functional Imaging, Tianjin Medical University General Hospital, Tianjin, China; ^2^ Department of Neuropathology, Tianjin Neurological Institute, Tianjin Medical University General Hospital, Tianjin, China

**Keywords:** skull-base, multifocal paraganglioma, carotid body tumor, dopamine, 3-methoxytyramine

## Abstract

**Background:**

Bilateral carotid body tumors with a concomitant skull-base paraganglioma are extremely rare, of which only one case has been reported in the literature to date.

**Case presentation:**

We present the case of a 35-year-old male with 1 year of hypertension and high levels of dopamine and 3-methoxytyramine. Magnetic resonance imaging (MRI) scans demonstrated three separate masses at the left middle cranial fossa floor and bilateral carotid bifurcation. Genetic testing showed succinate dehydrogenase complex subunit D mutation. The patient underwent the resection of the left skull base mass. Histopathology and immunohistochemistry confirmed the presence of a skull-base paraganglioma.

**Conclusions:**

Succinate dehydrogenase complex subunit D mutation-associated bilateral carotid body tumors with a concomitant skull-base paraganglioma accompanied by abnormal dopamine and hypertension are extremely rare, which not only provides ideas for considering the association of gene mutations, biochemical abnormalities and clinical symptoms but also provides an expanded diagnostic spectrum for paraganglioma in atypical locations.

## Introduction

Paragangliomas (PGLs) are rare slow-growing highly vascular tumors that arise from paraganglia, which are derived from cells of neural crest origin, dispersed in various areas throughout the body (1). Approximately 30% to 40% of PGLs have a genetic factor, most commonly due to mutations in the gene encoding succinate dehydrogenase enzyme complex (SDH) (2), among which SDHD mutations are mainly associated with multifocal head and neck paraganglioma (HNPGL) and benign PGL (3). But the form of bilateral carotid body tumors with a concomitant skull-base PGL is extremely rare, of which only one case has been reported in the literature to date (4).

HNPGLs are nonfunctional generally, but excessive dopamine or 3-methoxytyramine (3-MT) in urine or plasma has been demonstrated in 19–28% of patients with a HNPGL (5–7). Catecholamine-synthesizing enzymes, in particular aromatic L-amino acid decar-boxylase (AADC), which promotes the conversion of L-3,4-dihydroxyphenylalanine (L -DOPA) to dopamine, are expressed in the majority of HNPGL tissues (8), such as carotid body PGLs (9). Dopamine production has been shown to be particularly prevalent among SDHD mutation carriers (8).

Here, we report such a case that the patient as a SDHD mutation carrier had bilateral carotid body tumors with a concomitant skull-base PGL and presented high levels of plasma dopamine and 3-MT.

## Case report

**Figure 1:**
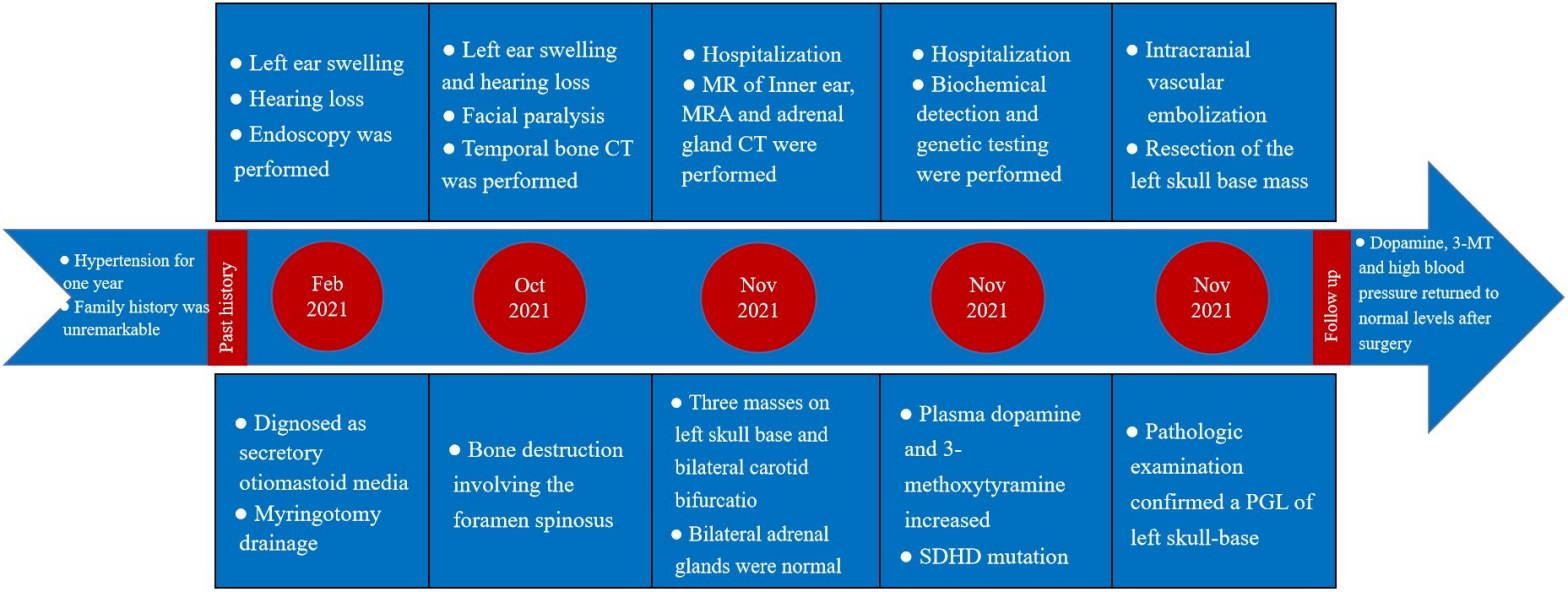
Case treatment process timeline.

**Figure 2:**
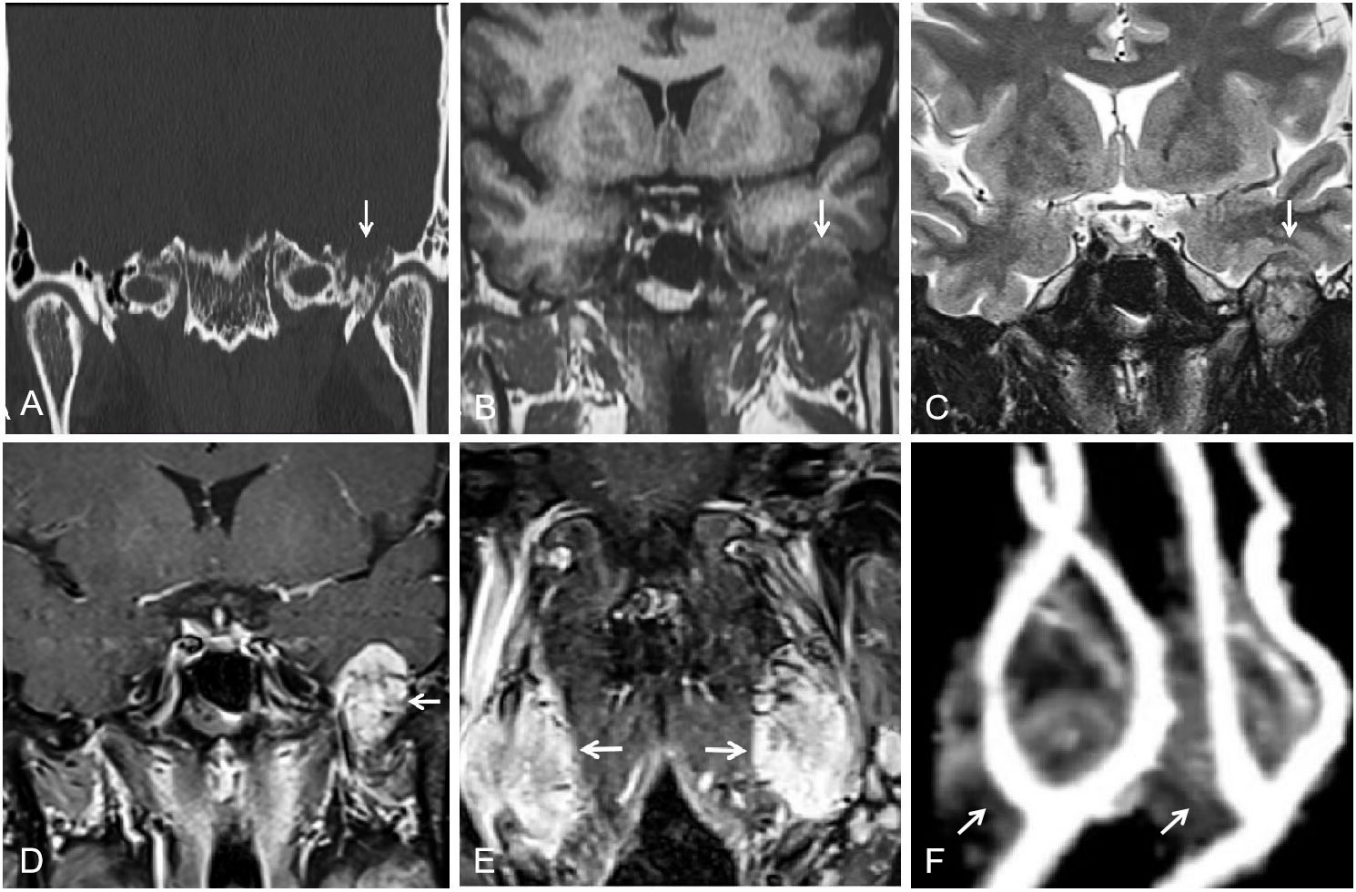
**(A)** Temporal bone CT showed permeative bone destruction involving the foramen spinosum at the left skull base (arrow). **(B, C)** T1WI and T2WI showed a slightly hyperintense peripheral to the left skull-base mass and a heterogeneous hypointense internal (arrow). **(D, E)** After contrast administration, masses of left skull base and bilateral carotid bifurcation showed intense enhancement with signal voids (arrow). **(F)** MRA showed a classic lyre sign on bilateral carotid bifurcation (arrow).

**Figure 3:**
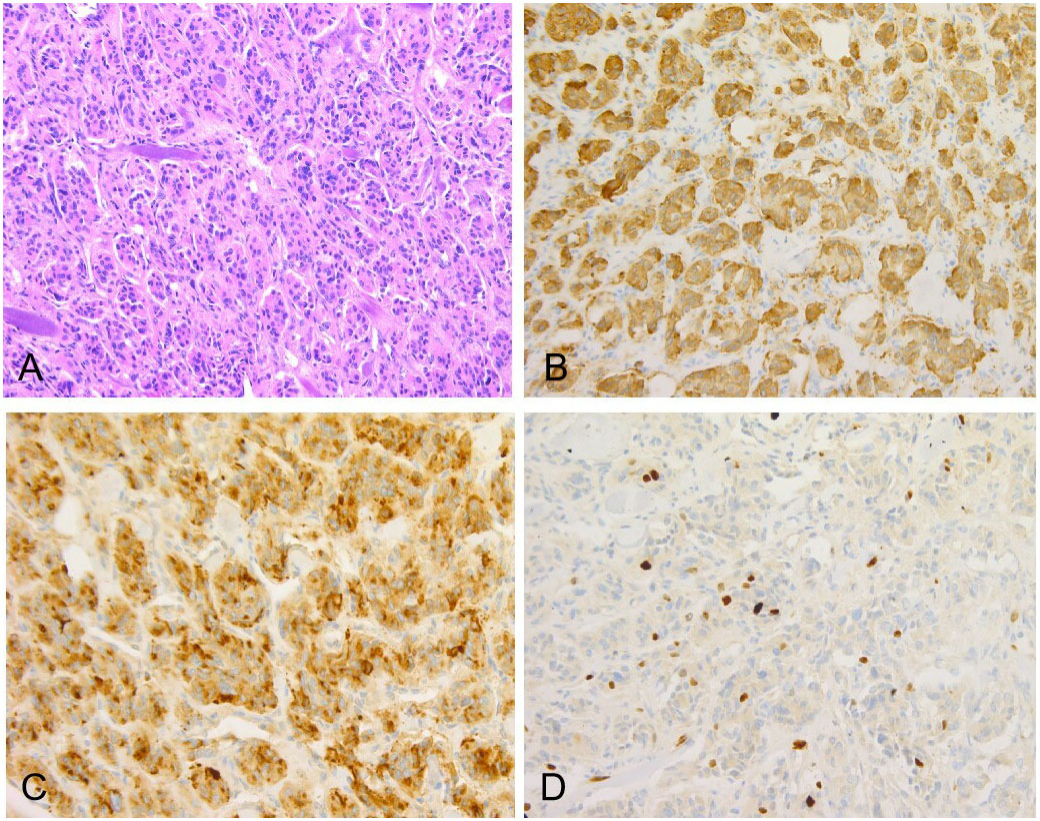
**(A)** Original magnification×100, showed the characteristic Zellballen morphology, collagen fibers distributed between nests of tumor cells (H&E). Immunostains for neuroendocrine markers including Syn **(B)** and CgA **(C)** highlighted chief cells. **(D)** The Ki67 proliferation index was 6.10%.

The patient’s treatment process in our hospital has been briefly shown through the timeline (**Figure 1**). Specifically speaking, a 35-year-old male patient was admitted to our institution with left ear pain accompanied by hearing loss for more than one month. Three days ago, facial paralysis occurred, accompanied by left facial swelling. His plasma levels of dopamine and 3-methoxytyramine increased significantly, which were more than 20 times and 3 times higher than normal, respectively. The patient was previously diagnosed with secretory otiomastoiditis media and had myringotomy drainage. He also had a history of hypertension for one year, the highest was about 150/110mmHg, which could be controlled after taking anti-hypertensive drugs. His family history was unremarkable. MRI scans demonstrated three separate masses in the head and neck region. The first one was located at the left middle cranial fossa floor measuring approximately 2.0×2.1×2.3 cm. Temporal bone CT showed permeative bone destruction involving the foramen spinosum. T1-weighted imaging (T1WI) and T2-weighted imaging (T2WI) revealed hyperintense with foci of hypointense. After contrast administration, the mass showed intense enhancement with signal voids. The other two were located on each side of the carotid bifurcation measuring approximately 4.43×3.1 cm and 3.89×2.54 cm respectively and demonstrated the same enhancement patterns as the mass of the left temporal bone did. MR angiography (MRA) confirmed a classic lyre sign on bilateral carotid bifurcation (**Figure 2**). Thus, the most likely diagnosis of the three masses was multiple PGLs based on the classic radiologic feature. No definite abnormalities were found in bilateral adrenal glands. Given the multifocal nature of the patient’s PGLs, there is a significant chance that this case is an inherited disease. So genetic testing is necessary for the patient and the results showed SDHD mutation. Later, the patient first underwent intracranial vascular embolization, during which it was found that the tumor was clearly stained with contrast media, and was supplied by the left middle meningeal artery and its branches. Then the part of the supplying arteries were embolized. Subsequently, the resection of the left skull base mass was performed, it was found that the mass was gray, tough, and located outside the dura. Although part of the supplying arteries had been embolized, there was significant intraoperative bleeding. The mass was completely excised. Pathologic examination confirmed the diagnosis of a PGL with positive immunostaining for synaptophysin (Syn) and chromogranin A (CgA). Microscopically, hematoxylin and eosin (H&E) staining of the tissue sections revealed a characteristic nested architectural pattern (Zellballen). The Ki67 labelling index (Ki67 LI) was 6.10% (**Figure 3**). According to histological pattern, cellularity, comedo-type necrosis, capsular/vascular invasion, Ki67 LI and catecholamine type, the grading system for adrenal phaeochromocytoma and paraganglioma (GAPP) score of this PGL is 4 points. The patient recovered well after surgery. Head and neck MR and adrenal CT followed up regularly. The patient’s carotid body tumors showed no significant changes in imaging, and no abnormalities were observed in adrenal and retroperitoneal areas. Dopamine, 3-MT and blood pressure were back to normal range. Since the patient’s carotid body tumors were considered to be non-functional, and the patient did not present with symptoms related to carotid artery compression by the carotid body tumor, the clinician and patient agreed to monitor bilateral carotid body tumors only for the time being.

## Discussion

In this case, the multifocal HNPGL mediated by SDHD mutation were distinctive in the form of bilateral carotid body tumors with cranial base PGL.

HNPGLs lack the characteristic norepinephrine and epinephrine production of pheochromocytomas usually (8), but the literature reports that at least 20% of cases are associated with dopamine hypersecretion (10). The four subunits of SDH was encoded by SDHA, - B, - C and - D genes, which together form the mitochondrial complex II. A mutation in one of these subunits has an inhibitory effect on prolyl hydroxylase domain proteins and diminishes the degradation of hypoxia-inducible factor α. This might have an effect on the dopamine secretion (3, 8). Dopamine, 3-MT and high blood pressure all returned to normal range after surgery in this case, therefore, we speculate that this skull PGL was a functional tumor, and that the excess dopamine and 3-MT produced by it,rather than carotid body tumors, led to hypertension.

As what was mentioned before, multifocal HNPGL, associated with SDHD mutations (3), typically occurs at sites rich in paraganglioma cells (1). However, the skull base mass in this case was located at the foramen spinosum region, where paraganglioma cells are absent. It is supposed that these PGLs in rare locations far away from the specific organs may be related to the abnormal migration of paraganglioma cells, which are more widely distributed in fetuses than in adults (11). All PGLs have the potential to metastasize (12). GAPP score of this PGL is 4 points, indicating that the tumor is moderately differentiated (MD) type. 60% of MD tumors metastasized, compared with fewer than 4% of well-differentiated type tumors (13). It is generally accepted that PGL with high Ki67 LI highly metastasize (13), Ki67 LI more than 6% in this case is less common. Dopamine hypersecretion was considered a feature of immaturity (14), it is reported that the plasma level of Methoxytyramine, the metabolite of dopamine, is 4.7-fold higher in patients with metastases than in those without, suggesting its use as a potential biomarker (5). Rare cases of SDHD-associated metastatic HNPGLs have also been described (15, 16). However, generally speaking, the risk of metastasis of SDHD-associated PGLs is much lower than that of SDHB-associated PGLs (1, 2), similarly, parasympathetic PGLs are less likely to metastasize than sympathetic PGLs (12). Furthermore, if the skull base PGL of this patient had metastasized from the carotid body tumors, it should have the same biochemical characteristics as carotid body tumors do theoretically, which are nonfunctional parasympathetic PGLs. We speculate the skull-base mass of our case is a primary parasympathetic PGL associated with excessive dopamine secretion that causes hypertension.

Additionally, the facial paralysis may be explained by a mass squeezing and narrowing the eustachian tube, leading to secretory middle ear mastoiditis, which further involved the facial nerve and resulted in peripheral facial paralysis. As predicted, the symptoms were totally relieved after surgery.

This case report has been approved by the patient. The patient stated that his initial goal was to relieve facial paralysis, otitis media and other symptoms. After relevant examinations, the patient agreed with the doctor’s preliminary diagnosis of multiple paragangliomas and agreed that the above symptoms are likely to be caused by them. During hospitalization, the patient was aware of and agreed with the clinician’s treatment schedule for partial intracranial vascular embolization and resection of the left skull base tumor. After the surgery, symptoms were relieved, so the patient was positive about the surgical treatment. Due to the good recovery and no other discomfort so far, the patient also agreed to have periodic review of bilateral carotid body tumors for the time being, and to undergo surgery if relevant symptoms appear. In addition, the patient was also aware that the disease may be genetically related and has the potential to metastasize, so the patient and his family indicated that they would follow the doctor’s advice to regularly follow up the head and neck MR and abdominal CT.

## Conclusions

SDHD mutation-mediated bilateral carotid body tumors with a concomitant skull-base PGL, leading to excess dopamine and 3-MT secretion and subsequent hypertension, are extremely rare, which not only provides ideas for considering the association of gene mutations, biochemical abnormalities and clinical symptoms, so as to carry out targeted examination, but also provides an expanded diagnostic spectrum for PGLs in atypical locations. But due to biological inactivity of patient’s bilateral carotid body tumors, they are most probably not considered for histopathological verification. This represents a weak point of our case report.

## Data availability statement

The original contributions presented in the study are included in the article/supplementary material. Further inquiries can be directed to the corresponding author.

## Ethics statement

The studies involving human participants were reviewed and approved by the Research Ethics Committee of Tianjin Medical University General Hospital. The patients/participants provided their written informed consent to participate in this study. Written informed consent was obtained from the patients/participants for the publication of any potentially identifiable data/images included in this article.

## Author contributions

ZL: medical history data collection, radiological investigation and follow-up, drafting and revising the manuscript, final approval, and agreement to be accountable. RY: medical history data collection, radiological investigation, final approval, and agreement to be accountable. CS: pathological and immunohistochemical examination, final approval, and agreement to be accountable. JW: contribution to study design, revising the manuscript, final approval, and agreement to be accountable. All authors contributed to the article and approved the submitted version.
